# Implantable cardioverter-defibrillator in a patient with dextrocardia situs inversus

**Published:** 2016

**Authors:** Mehrdad Saravi, Rozita Jalalian, Mohamadtaghi Hedayati

**Affiliations:** 1 Cardiology Department, Babol University of Medical Sciences, Babol, Iran.; 2 Cardiology Department of Mazandaran University of Medical Sciences, Sari, Iran.

**Keywords:** Dextrocardia, Situs inversus, Pacemaker, Implantable cardioverter-defibrillator (ICD), Multislice computed tomography

## Abstract

**Background:**

Dextrocardia is a congenital anomaly, which may have coexistent coronary artery disease (CAD), arrhythmias and conventional indications for device therapy. However, the implantation of transvenous leads can be technically challenging and the approach needs to be tailored to the patient's individual anatomy.

**Case presentation:**

A 54-year-old male with dextrocardia situs inversus and ischemic left ventricular dysfunction developed ventricular tachycardia and fibrillation. Therefore, left- sided approach, dual chamber implantable cardioverter-defibrillator (ICD) was applied using a conventional method and standard equipment after complete evaluation of cardiac anatomy and vascular assessment.

**Conclusion:**

Electrical device implantation in patients with dextrocardia is possible after obtaining complete information about anatomy and/or coexisting congenital abnormalities, which helps in obtaining appropriate implantation approach.

Dextrocardia is a congenital abnormality (1) in which the heart is located in the right hemithorax, due to an abnormal embryological cardiac development. It is rare, with a prevalence - 0.4 per 10,000 live births. Approximately one-third of the cases are associated with situs inversus, where the major visceral organs, including the heart, are a mirror image of their normal position. Although dextrocardia with situs inversus may occur in association with other congenital cardiac anomalies (only 3%) (2), it can be an isolated finding with normal life expectancy (3). Dextrocardia must be distinguished from dextroposition, which is defined as the presence of the heart in the right hemithorax with normal alignment of the major heart axis (5). Patients with congenital heart disease may have coexistent CAD and conventional indications for device therapy. However, the transvenous lead implantation can be technically challenging and the approach needs to be tailored to the patient's individual anatomy (5, 6). There are some reports about electrical cardiac device implantation but most of them are related to pacemakers and a few ones are related to ICD implantation. 

## Case presentation

A 54-year-old male with dextrocardia true situs inversus, referred to our hospital due to traumatic syncope. He had history of coronary artery bypass grafting 4 months ago, and inferior wall myocardial infarction 3 months ago, as a result, he was not a candidate for any revascularization. 

**Figure 1:**
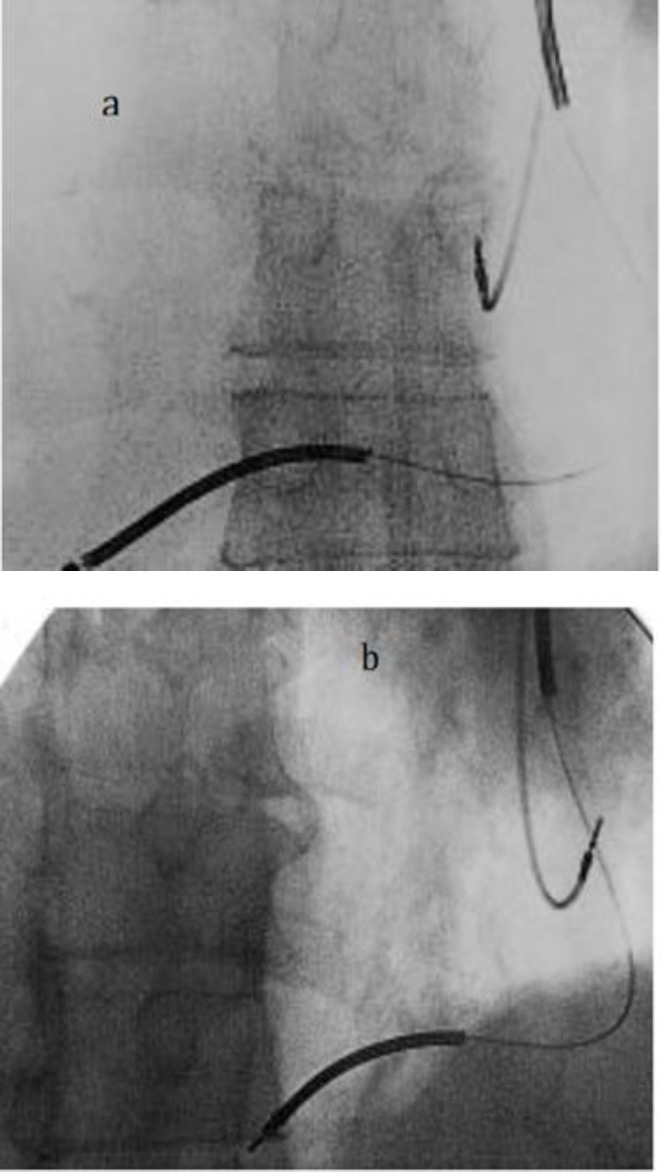
Fluoroscopic images obtained in anteroposterior view and 30 ^°^ left and right anterior oblique views a and b respectively

He had two attacks of syncope due to sustained monomorphic and polymorphic ventricular tachycardias and left ventricular ejection fraction of 35% by an echocardiogram done three weeks ago. Since he was not a candidate for further revascularization, we decided to implant a dual-chamber implantable cardioverter-defibrillator (ICD) (7). Before procedure, bilateral axillary venography, fluoroscopy, multislice computerized tomography angiography (MSCTA) were done which revealed dextrocardia with normal segmental cardiac anatomy of chambers and vascular bed. Venous access was gained via the left subclavian vein. Through a left-sided superior vena cava, two active leads were implanted in right ventricular apex (3830-59, Medtronic, Minneapolis, USA) and right atrial (5568 Medtronic, Minneapolis, MN, USA) appendage. Figure 1 shows the final position of the leads. The thresholds and senses were located appropriately. The leads are connected to a double-chamber ICD. Three months later, cardiovascular assessment of the patient was acceptable. 

## Discussion

We implanted an ICD with left-sided approach with conventional method in a patient with dextrocardia situs inversus, ischemic left ventricular dysfunction and life threatening ventricular arrhythmias. Dextrocardia must be distinguished from dextroposition, which is defined as the presence of the heart in the right hemithorax with normal alignment of the major heart axis (4). Implantation of device in this group of patients is challenging and related to the patient's individual anatomy (5, 6). Information about anatomy and/or coexisting congenital abnormalities, which may preclude percutaneous approach should be obtained before procedure (8). Dextrocardia has some additional difficulties like extra angle imposed on the leads when passing through the abnormal course of superior vena cava and reversed position of right atrium and right ventricle that lies in the variable anatomy itself imaging techniques, including multislice computed tomography, targeted to pacing system and unusual anatomical relationships are important before implantation (9, 10).

0Information about anatomy and/or coexisting congenital abnormalities, which may preclude percutaneous approach should be obtained before procedure (1, 11, 12). Fluoroscopy may be helpful in delineation of the chambers but with different appearance and sometimes of limited value (13, 14). Fluoroscopic images (f) are presented in anteroposterior view, 30º left anterior oblique view and 30º right anterior oblique view (from left to the right). Figure: Fluoroscopic images obtained in anteroposterior view, 30º left anterior oblique view and 30º right anterior oblique view (11). To overcome these difficulties, it may be helpful to invert the fluoroscopic image left-to-right to simulate normal anatomy, and more operation time is likely needed to successfully place leads in optimal positions (3). Since anomaly of venous system is commonly associated with mirror-image dextrocardia, angiogram is necessary prior to permanent pacemaker implantation (15, 16). 

Magnetic resonance imaging may reveal important information on both issues (17)^. ^Cardiac computed tomographic (CT) angiography was performed as a road to implantation procedure (18). Some prefer right-sided approach because of placing generator to the left side of the chest which may result in high defibrillation thresholds or even insufficient defibrillation because of the fact that the critical myocardial mass lies on the left side (11, 19).

## References

[1] Goyal SL, Lichestein E, Gupta PK, Chadda KD, Lajam F (1976). Sick sinus syndrome requiring permanent pacemaker implantation in a patient with mirror-image dextrocardia. Chest.

[2] Fang Y, Jiang LC, Chen M (2009). Successful pacemaker implantation in a patient with dextrocardia situs inversus totalis. Europace.

[3] Bohun CM, Potts JE, Casey BM, Sandor GGS (2007). A population-based study of cardiac malformations and outcomes associated with dextrocardia. Am J Cardiol.

[4] Abbas AE, Liu P, Lee RW (2004). Acquired post-pneumonectomy dextrocardia. Interact CardioVasc Thorac Surg.

[5] Paul A Scott, Paul R Roberts (2009). Cardiac resynchronization therapy upgrade in a patient with dextrocardia and situs inversus. Europace.

[6] Badui E, Lepe L, Solorio S, Sánchez H (1995). Heart block in dextrocardia with situs inversus A case report. Angiology.

[7] Epstein AE, DiMarco JP, Ellenbogen KA (2008). AHA Practice Guideline: Executive Summary ACC/AHA/HRS 2008 Guidelines for Device-Based Therapy of Cardiac Rhythm Abnormalities: Executive Summary. Circulation.

[8] Pott C, Brar R, Valderrábano M (2006). Implant of a biventricular pacemaker in patient with dextrocardia and persistent left superior vena cava. Pacing Clin Electrophysiol.

[9] Małecka B, Bednarek J, Tomkiewicz-Pająk L (2010). Resynchronization therapy transvenous approach in dextrocardia and congenitally corrected transposition of great arteries. Cardiol J.

[10] Brito MR, Miranda CE, Barros VC, Castro LR, Borges MH (2001). Heart block in dextrocardia and situs inversus: a case report. Ann Noninvasive Electrocardiol.

[11] Aliyev F, Turkoglu C, Uzunhasan I, Celiker C (2010). Periprocedural considerations during implantation of ICD in a patient with dextrocardia. Indian Pacing Electrophysiol J.

[12] Bindra PS, Lin D, Brozena S, Marchlinski F, Dixit S (2006). Case report: Placement of a coronary sinus lead in a patient with dextrocardia and situs inversus. J Interv Card Electrophysiol.

[13] Hamm K, Haghi D, Borggrefe M, Kuschyk J (2010). Challenging pacemaker implantation in a patient with acquired dextrocardia after pneumonectomy, skoliosis and complete heart block. Interact CardioVasc Thorac Surg.

[14] Subbiah RN, Gula LJ, Yee R (2007). Images in cardiovascular medicine Pacemaker implantation in a patient with dextrocardia, corrected transposition, and situs inversus. Circulation.

[15] Koyama K, Suzuki S, Fukui K (1993). Transvenous pacemaker implantation for sick sinus syndrome with mirror-image dextrocardia. Kokyu To Junkan.

[16] Słowiński S, Derlaga B, Kapusta J (1990). Permanent cardiac stimulation in a patient with isolated dextrocardia and ventricular septal defect. Pol Tyg Lek.

[17] Zartner PA, Wiebe W, Volkmer M, Thomas D, Schneider M (2009). Transvenous cardiac resynchronization therapy in complex congenital heart diseases: dextrocardia with transposition of the great arteries after Mustard operation. Europace.

[18] Al Fagih A, Al Najashi K, Dagriri K, Al Otay A, Al Ghamdi SA (2010). Feasibility of cardiac resynchronization therapy in a patient with complex congenital heart disease and dextrocardia, facilitated by cardiac computed tomography and coronary sinus. Venography Hellenic J Cardiol.

[19] Gold MR, Shin HT, Herre J (2007). Low Energy Safety Study Investigators Comparison of defibrillation efficacy and survival associated with right versus left pectoral placement for implantable defibrillators. Am J Cardiol.

